# A Cell-Permeant Nanobody-Based Degrader That Induces Fetal Hemoglobin

**DOI:** 10.1021/acscentsci.2c00998

**Published:** 2022-12-14

**Authors:** Fangfang Shen, Ge Zheng, Mekedlawit Setegne, Karin Tenglin, Manizheh Izadi, Henry Xie, Liting Zhai, Stuart H. Orkin, Laura M. K. Dassama

**Affiliations:** † Department of Chemistry and Sarafan ChEM-H, Stanford University, Stanford, California 94305, United States; ‡ Dana Farber Boston Children’s Cancer and Blood Disorders Center and Howard Hughes Medical Institute, Boston, Massachusetts 02215, United States; § Department of Pediatrics, Harvard Medical School, Boston, Massachusetts 02115, United States

## Abstract

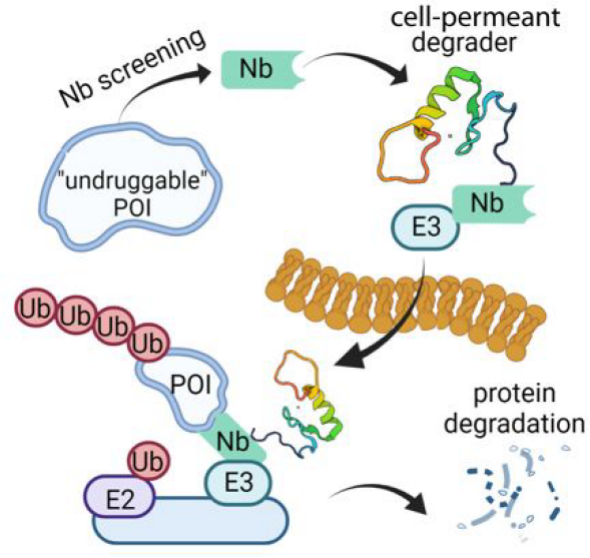

Proximity-based strategies to degrade proteins have enormous therapeutic potential in medicine, but the technologies are limited to proteins for which small molecule ligands exist. The identification of such ligands for therapeutically relevant but “undruggable” proteins remains challenging. Herein, we employed yeast surface display of synthetic nanobodies to identify a protein ligand selective for BCL11A, a critical repressor of fetal globin gene transcription. Fusion of the nanobody to a cell-permeant miniature protein and an E3 adaptor creates a degrader that depletes cellular BCL11A in differentiated primary erythroid precursor cells, thereby inducing the expression of fetal hemoglobin, a modifier of clinical severity of sickle cell disease and β-thalassemia. Our strategy provides a means of fetal hemoglobin induction through reversible, temporal modulation of BCL11A. Additionally, it establishes a new paradigm for the targeted degradation of previously intractable proteins.

## Abstract 2

A cell-permeant, protein-based degrader is used to induce fetal hemoglobin in erythroid precursor cells through temporal proteasomal degradation of BCL11A.

## Introduction

Proteolysis targeting chimeric molecules (PROTACs) and molecular glue degraders, such as the immunomodulatory imide drugs, hijack the cellular protein ubiquitination machinery to specifically degrade proteins of interest (POIs).^1,2^ They offer exciting opportunities for use as therapeutics and serve as powerful research tools for biological inquiry.^3,4^ While PROTAC molecules are being used clinically with notable success and great promise,^5^ it remains challenging to target many therapeutically relevant protein families due to challenges inherent to ligand discovery and optimization. This is especially true for transcription factors (TFs), which generally contain unstructured domains and lack obvious “ligandable” pockets.^6,7^ Additionally, the development of PROTACs requires a substantial synthetic effort to test various combinations of recruited ubiquitin E3 ligases and linkers that are optimal for forming a ternary complex of the PROTAC, POI, and ubiquitin E3 ligase.^8^ Other degradation platforms, including the degradation tag system^9^ and transcription factor targeting chimeras,^10^ have been developed for difficult protein targets. However, the utility of these platforms is either limited to engineered proteins, or their specificity for the targeted proteins remains unexplored.

Unlike the small molecule ligands that are typically used in PROTACs, antibodies exploit features of protein surfaces to recognize antigens and show exceptional specificity and remarkable affinity for their antigens. Even fragments of single variable heavy chain domains, termed nanobodies (Nbs), retain antigen specificity and can be used as the POI ligand in a PROTAC. The recent development of a yeast surface display platform to screen large libraries of synthetic nanobodies provides a straightforward and low-cost method to obtain Nb ligands for proteins.^11^ While protein-based degraders have been explored,^12−18^ their potential is hindered either by the challenge of delivering these ligands to intracellular targets or because the ligands do not target endogenous proteins. We hypothesized that appending a cell-penetrating moiety to Nb degraders could overcome these limitations and provide a broad strategy to degrade endogenous proteins, including poorly structured targets for which small molecule ligands do not exist. To exemplify the approach, we focused on the transcription factor BCL11A because it is a clinically validated target for the treatment of hemoglobin disorders, including β-thalassemia and sickle cell disease (SCD).^19,20^

Reactivation of fetal hemoglobin (HbF, α_2_γ_2_) is a promising strategy to ameliorate clinical severity in patients with hemoglobin disorders.^21^ Patients with SCD who produce elevated HbF exhibit significantly improved survival rates.^22^ BCL11A represses HbF expression through direct binding at the γ-globin promoters, eliciting the switch from fetal to adult hemoglobin (HbA, α_2_β_2_) expression during erythropoiesis.^19,23,24^ Genetic approaches, notably clustered regularly interspaced short palindromic repeats (CRISPR)–Cas9^25,26^ editing and RNA interference,^27^ have been used to downregulate BCL11A in patients and validated the clinical utility of disabling BCL11A. However, the resource-intensive care and high cost of ex vivo genetic manipulation of cells in clinical trials limit the application of these treatments. PROTACs may provide an alternative means to deplete BCL11A in a temporal, reversible, precise, and cost-effective manner.

PROTAC development for BCL11A faces a central challenge in that there is no available small molecule ligand specific for the protein. This is largely due to the vast amount of predicted structural disorder and the protein’s high similarity to a paralog, BCL11B. To overcome this challenge, we first identified protein-based ligands using a library of synthetic nanobodies displayed on the surface of yeast cells. Expression of top hits from the screen fused to the Fc domain of Immunoglobulin G1 led to Trim21 mediated loss of BCL11A but not its paralog BCL11B, indicating that the ligands are specific. Further functionalization of a top-performing candidate for cell penetration and E3 ligase recruitment created a cell-permeant, protein-based degrader for the degradation of endogenous BCL11A. Moreover, loss of BCL11A in response to treatment with the degrader resulted in a significant induction of fetal hemoglobin. While this degrader may advance efforts to modulate BCL11A, the strategies described here can be employed to create the cell-permeant protein-based degraders that control the levels of other similarly intractable proteins.

## Results

### Target Selection and Ligand Screen

**Figure 1:**
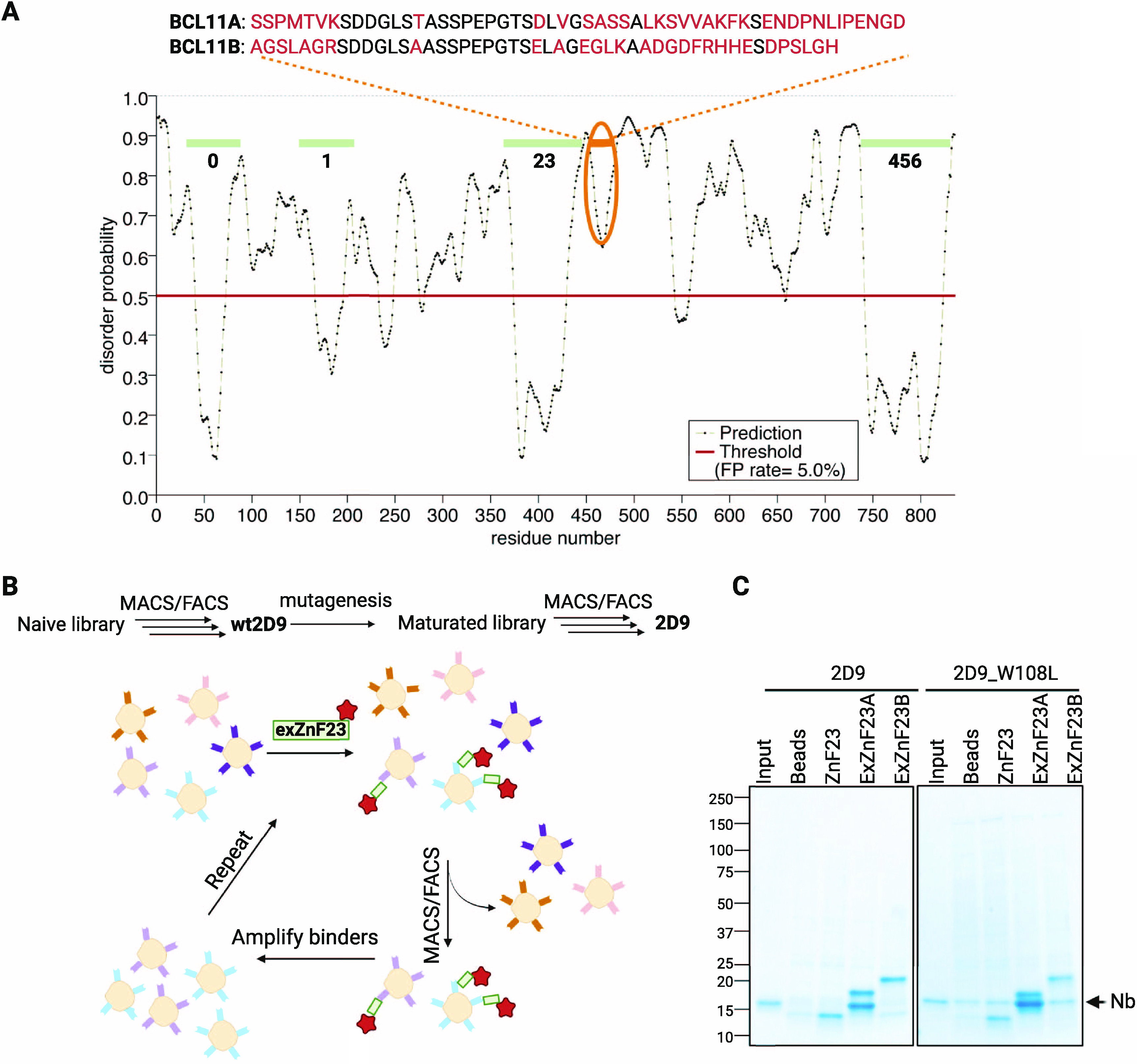
Identification and construction of cell permeant Nb ligands for BCL11A. (A) Disorder probability of BCL11A revealing predicted order in the ZnF domains (**0**–**6**) and the sequence divergence from BCL11B in the extended ZnF23 region. The ZnF23 domains of the paralogs are 93.2% identical and 96.6% similar; in the exZnF23 fragment, the sequence identity and similarity are 69.3% and 78.1%, respectively. (B) Flowchart summarizing Nb selection. (C) SDS-PAGE analysis showing that Nb **2D9** and **2D9_W108L** interact with exZnF23 of BCL11A (exZnF23A) but not ZnF23 and exZnF23 of BCL11B (exZnF23B).

Although BCL11A is predicted to be largely unstructured, the protein contains several well-ordered regions, including a CCHC-type zinc finger domain (ZnF0) that might mediate self-association^28^ and six C_2_H_2_-type zinc finger domains (ZnF1, ZnF23, and ZnF456) (Figure 1a). We reasoned that such well-folded domains could be used to identify Nb ligands for BCL11A. Because of the close sequence similarity between BCL11A and its paralog BCL11B in all of the zinc finger regions,^29^ ligands that bind to these domains might demonstrate affinity for both paralogs. With the aim of achieving specificity in ligands selected for BCL11A, we produced ZnF23 and an “extended” zinc finger domain (exZnF23), which includes the C-terminal unstructured region that diverges in sequence between the two paralogs (Figure 1a). The extended protein fragment, which is 69.3% identical to BCL11B (the ZnF23 fragment is 93.2% identical), was expressed in *Escherichia coli* and purified, and the recombinant protein was used in a screen of yeast surface display to identify synthetic Nbs binders (Figure 1b). An initial Nb hit (**wt2D9**) was produced in *E. coli* (Figure S1a), and its affinity for BCL11A was assessed using a pull-down assay (Figure S1b) and MicroScale Thermophoresis (MST, Figure S1c). Affinity maturation through random mutagenesis of **wt2D9** was performed, after which 7 Nbs with better affinities were obtained (Figure S2). Of these, **2D9_V102G** (hereafter referred to as **2D9**) and **2D9_W108L** were selected for additional studies because of their stability, high affinity, and specificity for exZnF23 of BCL11A (Figure 1c). Size-exclusion chromatography coupled with multiangle light scattering revealed that BCL11A exZnF23 is monomeric and formed a stable complex with **2D9** (Figure S3). A crystal of **2D9** complexed with exZnF23 of BCL11A was obtained, but insufficient electron density precluded modeling of BCL11A. Nonetheless, the high-resolution structure of **2D9** combined with maturation mutagenesis data suggested that some of the interaction with BCL11A is mediated through a loop in Complementarity Determining Region (CDR) 3 of the Nb (Figure S4).

### Functionalization of Ligands for Cell Penetration

**Figure 2:**
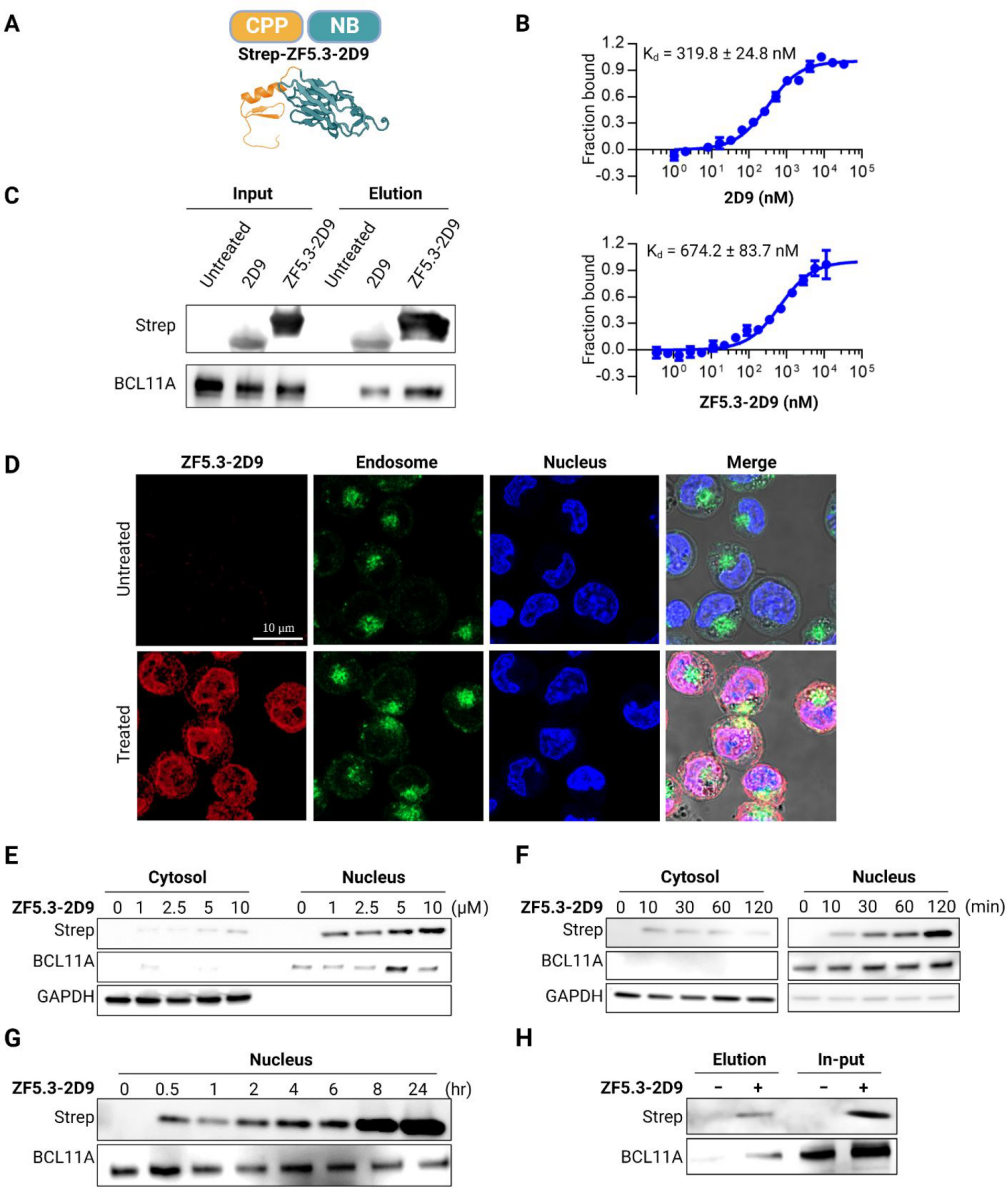
Delivery of **ZF5.3-2D9** to erythroid precursor cells. (A) Schematic description of the cell-permeant Nb **ZF5.3-2D9** created from the individual structures of ZF5.3 (modeled secondary structure) and **2D9** (PDB 7UTG). (B) MST analysis of the binding of **2D9** and **ZF5.3-2D9** to exZnF23 of BCL11A (mean ± SD, *n* = 3). (C) Immunoprecipitation revealing that **2D9** and **ZF5.3-2D9** bind to endogenous BCL11A. (D) Confocal microscopy images of HUDEP-2 cells revealing **ZF5.3-2D9** (red) entered HUDEP-2 cells and show significant colocalization (purple) with the nucleus (blue). Immunoblots revealing a (E) concentration- and (F) time-dependent cell penetration by **ZF5.3-2D9**. (G) Immunoblot showing increased accumulation of **ZF5.3-2D9** in the nucleus. (H) Co-immunoprecipitation of endogenous BCL11A using delivered **ZF5.3-2D9**. GAPDH and BCL11A were used as loading controls for the cytosolic and nuclear fractions, respectively.

Ligands intended for depletion of BCL11A must be delivered to the nucleus of erythroid precursor cells. Because Nbs are too large and unfavorably charged to traverse the plasma membrane, **2D9** was functionalized for cell penetration by appending a cell permeant miniature protein called ZF5.3^30−32^ (Figure 2a). MST measurements demonstrated that appending ZF5.3 to **2D9** did not significantly alter the affinity of **2D9** for BCL11A exZnF23 (Figure 2b). A pull-down assay using purified **2D9** or the fusion protein **ZF5.3-2D9** added into the lysate of human umbilical cord blood-derived erythroid progenitor (HUDEP-2) cells^33^ confirmed their association with endogenous, full-length BCL11A (Figure 2c). Cellular entry and protein localization of **ZF5.3-2D9** were first monitored by confocal imaging. Experiments in which **ZF5.3-2D9** was incubated with HUDEP-2 cells that were subsequently immunostained revealed accumulation of a significant fraction of the protein localized to the nucleus (Figure 2d). Immunoblotting demonstrated a dose- (Figure 2e) and time-dependent cellular uptake (Figure 2f), and cell fractionation showed that the fusion protein was largely present in the nucleus for at least 24 h (Figure 2g). Co-immunoprecipitation of BCL11A from HUDEP-2 cells incubated with **ZF5.3-2D9** further revealed its cellular entrance and binding to BCL11A (Figure 2h).

### Nanobody-Mediated Degradation of BCL11A

Having demonstrated that **ZF5.3-2D9** penetrated HUDEP-2 cells and engaged BCL11A, we used the cell-permeant Nb as a handle to mediate the proteasomal degradation of BCL11A. The rational design of PROTACs is difficult due to poor understanding of rules that govern formation of the ternary complex between the POI, ubiquitin E3 ligase, and the PROTAC. Unlike small molecule PROTACs, “all protein” degraders using reengineered E3 ligases are reported to exhibit high flexibility to various targets.^12,15^ However, these ligands are rarely cell permeant and do not degrade endogenous proteins, thereby limiting their utility.

**Figure 3:**
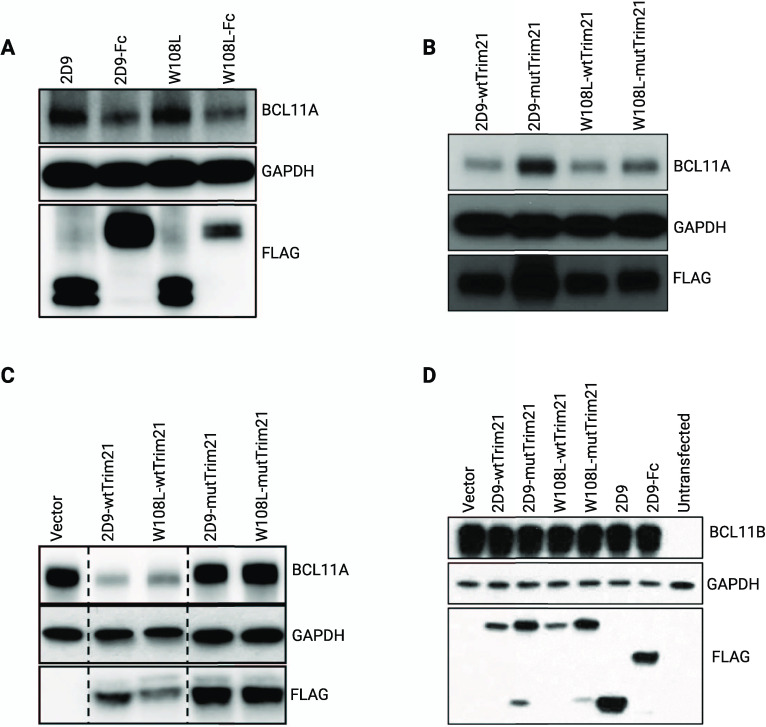
Nb **2D9** and **2D9_W108L** fused with Fc or Trim21 induced the degradation of BCL11A but not BCL11B. (A) Degradation of BCL11A upon lentiviral transduction of Nb-Fc fusion in differentiated HUDEP-2 cells. (B) Lentiviral transduction of Nb-wtTrim21 but not Nb-mutTrim21 led to degradation of BCL11A in HUDEP-2 cells. (C) Nb-wtTrim21 but not Nb-mutTrim21 led to degradation of overexpressed BCL11A in HEK293T cells. (D) Neither Nb-Fc nor Nb-Trim21 mediated degradation of overexpressed BCL11B in HEK293T cells.

To confirm that **2D9** can mediate selective degradation of BCL11A, we produced plasmids of the Nb fused to the Fc domain of Immunoglobulin G1 (Nb-Fc) or Trim21. With these designs, Trim21 would mediate proteasomal degradation via the Trim-Away method.^18^ As expected, lentiviral transduction of HUDEP-2 cells with Nb **2D9** or **2D9_W108L** did not affect BCL11A expression. However, transduction of **2D9-Fc** or **2D9_W108L-Fc** induced significant loss of BCL11A (Figure 3a). Similar experiments were performed with **2D9-wtTrim21**, **2D9_W108L-wtTrim21** and their corresponding variants (**2D9-mutTrim21** or **2D9_W108L-mutTrim21**). As shown in Figure 3b, Nb-wtTrim21 induced BCL11A downregulation, but no change was observed when mutTrim21 was used. To assess the specificity of the Nbs for BCL11A, we overexpressed BCL11A or BCL11B in HEK293T cells and transfected the cells with Nb-Fc and Nb-Trim21; as with HUDEP-2 cells, Nb-Fc and Nb-wtTrim21 induced loss of BCL11A. However, neither promoted loss of BCL11B (Figure 3c,d). Together, these data indicate that Nbs **2D9** and **2D9_W108L** can distinguish BCL11A from BCL11B and target endogenous BCL11A with high specificity.

**Figure 4:**
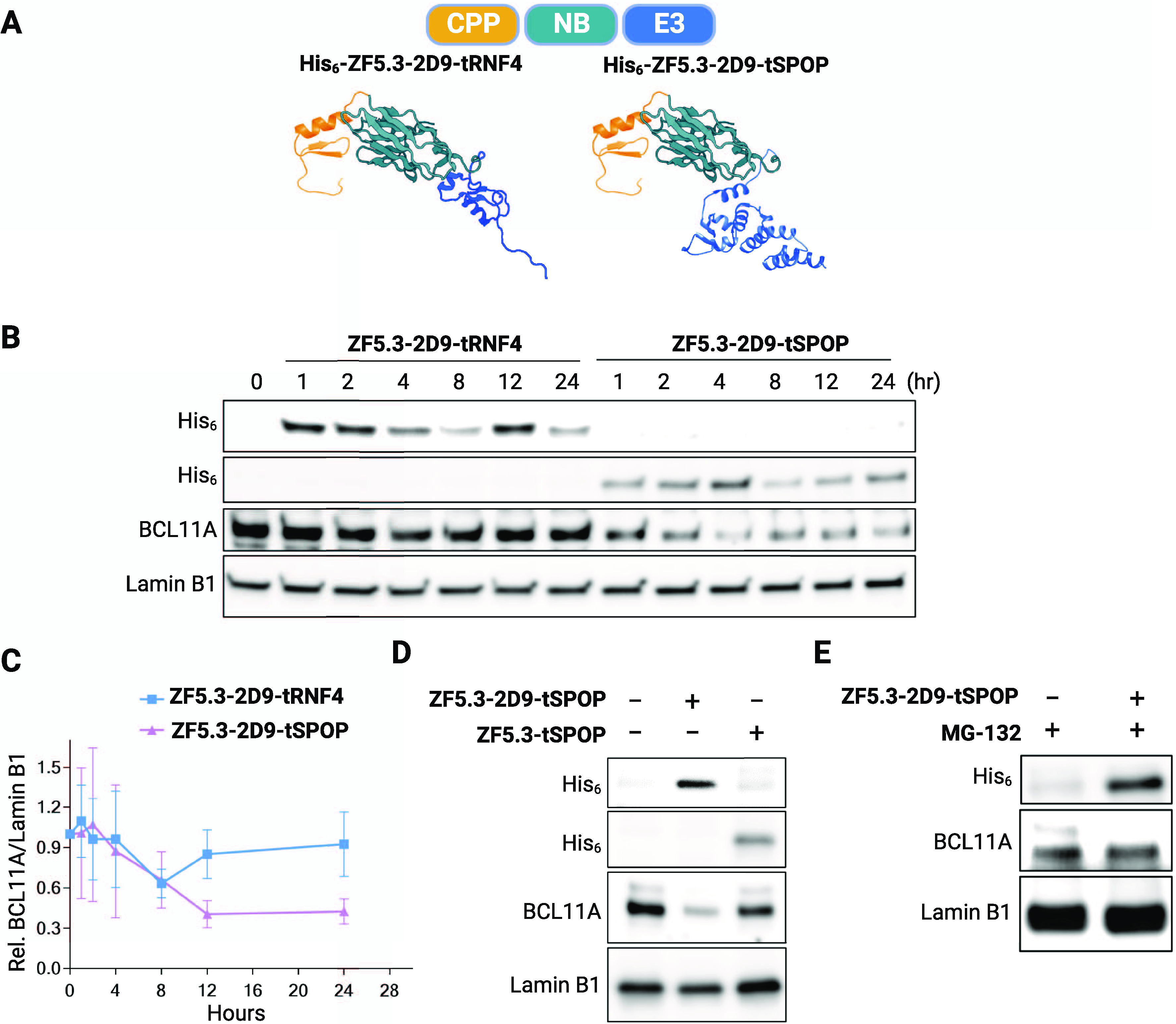
Nanobody-mediated degradation of BCL11A in HUDEP-2 cells. (A) Schematic depiction of BCL11A degraders. The individual structures of the RNF4 RING domain (4PPE), the BTB domain of SPOP (3HTM), ZF5.3 (modeled secondary structure), and **2D9** (7UTG) were retrieved from the Protein Data Bank. (B) Representative immunoblot showing loss of BCL11A in HUDEP-2 cells treated with **ZF5.3-2D9-tRNF4** or **ZF5.3-2D9-tSPOP**. (C) Quantification of BCL11A loss by immunoblots (mean ± SD, *n* = 3). Immunoblots revealing that (D) loss of BCL11A requires the presence of **2D9** and (E) is prevented by addition of 5 μM of the proteasome inhibitor **MG-132**. Lamin B1 was used as a loading control for the immunoblots.

Encouraged by these results, we designed cell-permeant, nanobody-based degraders for BCL11A by incorporating two ubiquitin E3 ligases into our design: engineered SPOP (speckle type POZ protein) and RNF4. These E3 ligases were chosen because of their high abundance in the nucleus and their previous use to mediate the degradation of nuclear proteins.^13,17^ SPOP is an E3 adaptor protein that functions in complex with cullin-3 (CUL3); it is composed of a substrate binding MATH domain and a CUL3-binding BTB domain. RNF4 is an E3 ligase that contains an N-terminal SUMO substrate binding site and a C-terminal RING domain responsible for dimerization and E2 binding. By replacing the native substrate recognition domain of SPOP and RNF4 with **ZF5.3-2D9**, we created the proteins **ZF5.3-2D9-tSPOP** and **ZF5.3-2D9-tRNF4** (Figure 4a). Both proteins were expressed and purified from *E. coli* (Figure S5) and were delivered in pure forms to HUDEP-2 cells through incubation (Figure S6). Upon their delivery, BCL11A levels were lowered steadily over time (Figure 4b, S7). With **ZF5.3-2D9-tSPOP**, the loss was striking, as up to 70% of BCL11A was depleted within 12 h of incubation with 10 μM of the degrader (Figure 4c). Moreover, the cell viability was minimally affected 24 h after treatment (Figure S8). Because of its far greater degradation effect than **ZF5.3-2D9-tRNF4**, **ZF5.3-2D9-tSPOP** was used for subsequent studies. With this degrader protein, loss of BCL11A in HUDEP-2 cells was sustained for at least 72 h (Figure S9). To exclude the possibility that the truncated SPOP delivered might have negative effects on the HbF repressive role of endogenous SPOP,^34^ we created the construct ZF5.3-tSPOP, where ZF5.3 was conjugated directly to the BTB domain of SPOP. In control experiments with ZF5.3-tSPOP, we verified that BCL11A levels remained unchanged (Figure 4d). Treatment with a proteasome inhibitor (MG-132) prevented the degradation of BCL11A, thereby confirming that the loss of the protein proceeded via proteasomal degradation in a ligand-dependent manner (Figure 4e).

### Fetal Hemoglobin Induction

**Figure 5:**
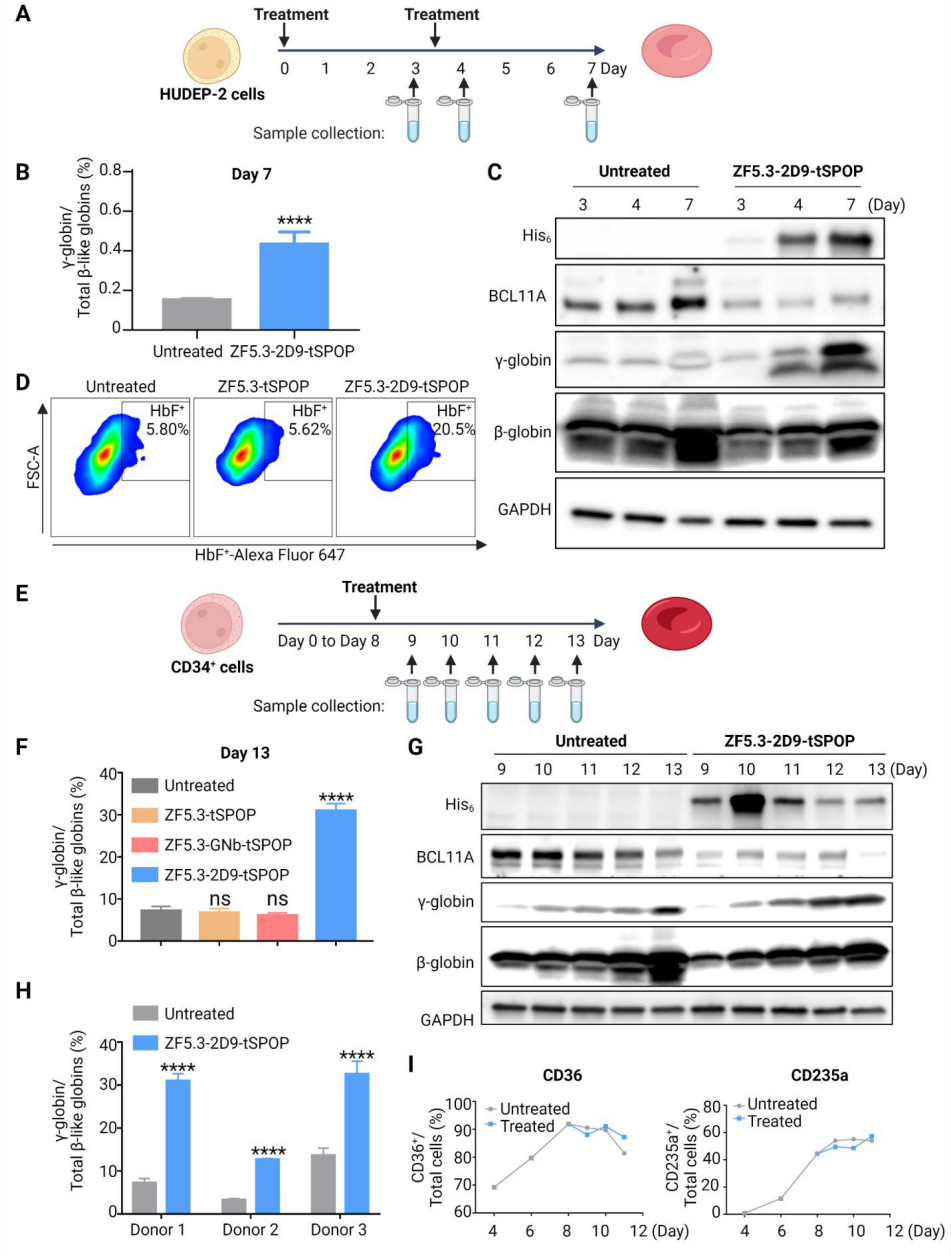
HbF induction in HUDEP-2 and CD34^+^ cells. (A) Schematic depiction of the HUDEP-2 cells differentiation and treatment. (B) qRT-PCR showing an increase in γ-globin mRNA after treatment with **ZF5.3-2D9-tSPOP** in HUDEP-2 cells (mean ± SD, *n* = 3, ****P < 0.0001). (C) Immunoblot revealing the loss of BCL11A and γ-globin increase after treatment with **ZF5.3-2D9-tSPOP** in HUDEP-2 cells. (D) Representative flow cytometric analysis of immunostained HUDEP-2 cells from Day 7 of differentiation showing an increase in the population of HbF^+^ cells following the degradation of BCL11A (*n* = 2). (E) Schematic depiction of the CD34^+^ cell differentiation and treatment. (F) qRT-PCR showing an increase of the γ-globin mRNA level after treatment with **ZF5.3-2D9-tSPOP** but not ZF5.3-tSPOP or ZF5.3-GNb-tSPOP in CD34^+^ cells (mean ± SD, *n* = 3, ****p < 0.0001). (G) Immunoblots revealing the loss of BCL11A and γ-globin increase after treatment with **ZF5.3-2D9-tSPOP** in CD34^+^ cells. GAPDH was used as a loading control in all immunoblots. (H) qRT-PCR showing an increase of γ-globin mRNA levels in CD34^+^ cells from three different donors after treatment with **ZF5.3-2D9-tSPOP** (mean ± SD, *n* = 3, ****p < 0.0001). (I) The population of CD36^+^ and CD235a^+^ in differentiating CD34^+^ cells treated with or without **ZF5.3-2D9-tSPOP**; two repeats in cells from different donors were performed.

Endogenous expression of BCL11A in HUDEP-2 cells varies during differentiation (Figure S10) and represses the expression of the fetal-stage γ-globin genes. Given the sustained loss of BCL11A in response to treatment with the degrader in undifferentiated HUDEP-2 cells, we explored its effect on differentiated HUDEP-2 cells. We first transduced HUDEP-2 cells with **2D9**, **2D9_W108L**, and their corresponding Fc/Trim conjugates (**2D9-Fc**, **W108L-Fc**, **2D9-Trim21**, and **W108L-Trim21**). The downregulation of BCL11A by Nb-Fc or Nb-Trim21 induced a significant increase of γ-globin transcripts in HUDEP-2 cells; no increase was observed for Nb only or Nb-mutTrim21 (Figure S11). Next, we investigated whether the cell-permeant degrader can also promote fetal hemoglobin induction. HUDEP-2 cells were treated with **ZF5.3-2D9-tSPOP** on Days 0 and 3 of differentiation, and samples were collected on Days 3 (prior the second treatment), 4, and 7 for analysis (Figure 5a). RT-qPCR of hemoglobin transcripts in samples from Day 7 (Figure 5b) revealed an increase of γ-globin transcripts in HUDEP-2 cells treated with **ZF5.3-2D9-tSPOP**. Immunoblots revealed that the degradation of BCL11A was maintained throughout and that significant fetal hemoglobin (HbF) was induced by Day 4 (Figure 5c). Fluorescence activated cell sorting of HbF-immunostained HUDEP-2 cells provided additional confirmation that treatment with **ZF5.3-2D9-tSPOP** resulted in a 3.5-fold increase of the HbF^+^ population, whereas treatment with ZF5.3-tSPOP, an effect similar to that of untreated cells (Figure 5d). These results further confirmed that the HbF reactivation observed is due to 2D9-dependent proteasomal degradation of BCL11A.

We further investigated the effect of **ZF5.3-2D9-tSPOP** in human primary CD34^+^ progenitor cells. The cells were cultured under differentiation conditions (Figure S12) and treated with **ZF5.3-2D9-tSPOP** on Day 8 of the differentiation (Figure 5e). RT-qPCR of hemoglobin transcripts in samples from Day 13 showed that upon treatment of **ZF5.3-2D9-tSPOP**, γ-globin increased to 30% of the total β-like hemoglobin. As observed with the experiments in HUDEP-2 cells, no induction of HbF was detected when CD34^+^ cells were treated with ZF5.3-tSPOP or with a second construct that replaced **2D9** with a Nb targeting GFP (ZF5.3-GNb-tSPOP, Figure 5f). Immunoblots of samples from Days 9–13 revealed sustained degradation of BCL11A over 5 days and marked HbF induction as of Day 11 (Figure 5g). As shown in Figure 5h, a range of a 2.4- to 3.9-fold increase in the levels of HBG transcripts was observed upon **ZF5.3-2D9-tSPOP** treatment in CD34^+^ cells from multiple donors, suggesting little donor-to-donor variability in globin induction. Additionally, treatment with the degrader did not affect the differentiation of CD34^+^ cells, as assessed by flow cytometric analyses of cell surface markers CD235a and CD36 (Figures 5i, S13). Although a similar percentage of viable differentiating CD34^+^ cells was observed, the cell proliferation rate was ∼2-fold slower in samples treated with the degrader when compared to control (Figure S14). This slower expansion effect is similar to what others have reported,^35^ and might be due to the loss of BCL11A as opposed to off-target toxicity. Regardless, these data together provide convincing evidence that a single treatment of the degrader **ZF5.3-2D9-tSPOP** is sufficient to permeate erythroid precursor cells, significantly deplete BCL11A, and elicit an increase in the expression of HbF.

## Discussion

Targeted protein degradation is attractive in that it has the potential to modulate proteins that have historically been intractable to conventional small molecules inhibitors. However, a central challenge remains: a small molecule ligand for the protein of interest must first be available to enable degrader synthesis. Because of this, a significant portion of the human proteome remains “undruggable”. Among these proteins are transcriptional regulators (activators and repressors), intrinsically disordered proteins, and scaffolding proteins. Successful modulation of these proteins with targeted protein degradation tools would invariably expand the scope of “druggable” human proteome. While difficult to be controlled with small molecules, many “undruggable” proteins contain surfaces to which proteins (or other large biomolecules) can bind, and these biomolecular ligands can be used as handles for protein modulation. In this work, we used a cell-permeant nanobody to degrade a traditionally undruggable protein. BCL11A was selected to demonstrate the utility of this approach because of its clinical significance for the β-hemoglobin disorders and because of its high sequence similarity to a close paralog, BCL11B. This work demonstrated that nanobodies can be tuned to recognize and differentiate the sequence differences of two paralogs, even if those differences occur in disordered regions. To further support the use of nanobodies for endogenous protein modulation, we engineered a BCL11A-specific Nb to create a nanobody-based degrader by incorporating cell penetration and E3 ligase recruitment moieties. Mere incubation of the degrader resulted in a sustained loss of BCL11A followed by a significant induction of fetal hemoglobin in primary erythroid precursor cells.

In conclusion, this work presents a systematic strategy for modulating the function of a traditionally “undruggable” target. If methods for efficient delivery of Nbs to cells *in vivo* can be developed, the degrader strategy described here could be considered for treatment of SCD and β-thalassemia. Apart from any clinical application, these studies establish a paradigm through which disease-relevant but intractable proteins can be targeted for modulation or other biological studies. Our strategy of creating a degrader by using a readily available nanobody library, a genetically encoded cell-penetrating vehicle, and an engineered E3 adapter protein significantly simplifies efforts to target other “undruggable” proteins.

## Data Availability

All data (except for the crystal structure deposited in the Protein Data Bank) are available in the manuscript or Supporting Information.

## Supporting Information Available

Detailed experimental procedures and protocols, additional data and figures including additional biochemical experiments, nanobody crystallography, additional flow cytometric analysis and immunoblots. Table S1: Primers and plasmids used for protein construction. Table S2: Proteins sequences. Table S3: Crystallographic data. Table S4, S5, and S6: Primers and Cq values for qPCR (PDF)
Transparent Peer Review report available (PDF)

## Accession Codes

The crystallographic structure for 2D9 has been deposited in the Protein Data Bank (PDB 7UTG).
