# Supplementary material for: A Cell-Permeant Nanobody-Based Degrader That Induces Fetal Hemoglobin
Source: ACS Cent Sci. 2022 Dec 14;8(12):1695–703. doi: 10.1021/acscentsci.2c00998 (PMC9801508; doi:10.1021/acscentsci.2c00998)
Supplement: Supplementary file 2 — oc2c00998_si_002.pdf [file oc2c00998_si_002.pdf]

oc-2022-00998u.R1

Name: Peer Review Information for "A cell-permeant nanobody-based degrader that induces fetal hemoglobin"

First Round of Reviewer Comments

Reviewer: 1

Comments to the Author

This is an excellent study from the Dassama lab developing a nanobody-based degrader for BCL11A to eliminate this protein to potentially reactive fetal global expression. The authors use yeast surface display to develop nanobodies that are specific for BCL11A. They show initial proof-of-concept of degradation by linking these nanobodies to a cell permeable miniature protein and either Fc domains to TRIM21 or to RNF4 or SPOP E3 ubiquitin ligases to deliver these biologic degraders to cells and effectively degrade BCL11A. BCL11A is a highly intractable disease target and developing a degrader strategy for this target using a fully small-molecule based approach is likely to be very challenging. The approach taken here is a big step towards developing a potential biologic-based degrader for BCL11A for hemoglobin disorders without having to rely on shRNA or CRISPR. This is a well-performed study and should be published as is.

Reviewer: 2

Comments to the Author

This manuscript presents a new approach to decrease BCL11A, a major regulator of fetal hemoglobin and one of the key therapeutic targets in sickle cell disease. While genetic ablation of BCL11A has been studied extensively and used in the clinical setting, after over a decade of study there has not been a successful drug approach to BCL11A inhibition or degradation. The cell-permeable nanobody-based approach to acutely degrade BCL11A presented in this paper provides a potentially useful tool for the study of BCL11A-mediated fetal hemoglobin repression. There are likely some significant limitations to the use of this approach in a therapeutic setting, but it may still be a valuable experimental tool. The biochemical data are well presented. The major critiques of this manuscript are regarding the validation experiments in erythroid cell lines and primary cells.

Major comments:

- For experiments in HUDEP and primary cells, it would be important to show data on whether these compounds affect cell viability and erythroid differentiation (by flow cytometry, expression of erythroid maturation markers, etc).

- Figure 5B: What do the error bars in this represent? Were replicates used? It would be more helpful to show change in gamma globin and beta globin transcripts, as well as the ratio. It's hard to interpret just the normalized data

- Figure 5D: The flow cytometry plots presented here are not typical of how the data are presented, and the gating for HbF positive versus negative cells looks very unclear. Again, there is no information on replicates presented anywhere.

- For the primary erythroid cell experiments presented in figure 5E-G, were cells from multiple donors tested? What do the error bars in figure 5F represent and how was significance determined? As the western blot quality is not very good, it would be helpful to have some quantification, again from multiple independent donors as biological replicates.

Minor points:

- Page 10, line 12-15 – the authors state that 30% gamma globin transcripts is sufficient to prevent sickling, but that threshold typically refers to HPLC measurements of globin proteins, not transcripts

- Supplemental Figure S2: I don't think "maturated" is the proper word

- "FACS" refers specifically to cell sorting, not analytical flow cytometry

Author's Response to Peer Review Comments:

Laura M. K. Dassama, Ph.D.
Assistant Professor of Chemistry
Assistant Professor of Microbiology & Immunology
Sarafan ChEM-H Institute Scholar

Phone: 650.723.1736
E-mail: dassama@stanford.edu

November 4, 2022

Dr. Editor
Senior Editor, ACS Central Science

Dear Dr. Editor:

We thank you for the handling of our manuscript, and the reviewers for their time and insightful comments. We have uploaded revised manuscript and supporting information files, as well as versions with changes tracked so that the changes are more readily identified by the editor and reviewers. Detailed responses to the reviewers are appended to this letter.

We hope that these modifications, along with those requested by the editorial office, make the manuscript suitable for publication in ACS Central Science.

Sincerely and on behalf of all co-authors,

Laura M. K. Dassama, Ph.D.
Assistant Professor, Department of Chemistry
Assistant Professor, Department of Microbiology & Immunology
Institute Scholar, Sarafan ChEM-H
Stanford University

Stuart H. Orkin, M.D.
David G. Nathan Distinguished Professor of Pediatrics
Harvard Medical School
Investigator, Howard Hughes Medical Institute

Reviewer: 1

Recommendation: Publish in ACS Central Science without change.

Authors' comment: We thank this reviewer for their time, and for the favorable view of our work.

Reviewer: 2

Recommendation: Reconsider after major revisions noted.

Authors' comment: We thank the reviewer for their time and constructive suggestions, and for providing us an opportunity to revise our work.

Reviewer: For experiments in HUDEP and primary cells, it would be important to show data on whether these compounds affect cell viability and erythroid differentiation (by flow cytometry, expression of erythroid maturation markers, etc).

Authors: We measured the toxicity of our degrader on HUDEP-2 cells 24h after protein delivery and found no significant cell viability loss after treatment (Fig. S8). Given that the effect of the degrader is more pronounced in primary cells, we elected to use these cells to probe the effects on erythroid differentiation. The data are now presented in Fig. S13 and Fig. 5I. In summary, we observed no significant impact of erythroid differentiation, as evidenced by the similar levels of $CD36^+$ and $CD235a^+$ cells in the treated vs untreated groups. However, even though similar levels of viable differentiating $CD34^+$ cells were observed in both groups, the proliferation rate was approximately 2-fold lower in samples treated with the degrader (Fig. S14). The slower rate of proliferation upon BCL11A loss has been reported before (Luc, S.; Huang, J.; McEldoon, J. L.; Somuncular, E.; Li, D.; Rhodes, C.; Mamoor, S.; Hou, S.; Xu, J.; Orkin, S. H. Bcl11a Deficiency Leads to Hematopoietic Stem Cell Defects with an Aging-like Phenotype. *Cell Rep.* **2016**, *16* (12), 3181-3194) and is therefore unlikely to be related to off-target toxicity of the degrader.

Reviewer: What do the error bars in this represent? Were replicates used? It would be more helpful to show change in gamma globin and beta globin transcripts, as well as the ratio. It's hard to interpret just the normalized data.

Authors: The bars represent are the mean values of 3 replicates while the error bars show the standard deviation from the mean. The Cq values from each replicate are provided in Table S5 and S6. Information on statistics is now added to the figure captions and in the SI methods. We have also modified the figure to show the change in globin transcripts rather than the ratio. Despite the larger value observed with $CD34^+$ cells, we note that the fold change is similar to that achieved with HUDEP-2 cells.

Reviewer: The flow cytometry plots presented here are not typical of how the data are presented, and the gating for HbF positive versus negative cells looks very unclear. Again, there is no information on replicates presented anywhere.

Authors: We have changed the gating look to match the convention used in the literature. We think that the data looks unclear because the transcript levels are small. HUDEP-2 cells are known tightly down-regulate HBG, which might contribute to the low levels observed. Despite the ~ 3-fold change in HbF^+ populations, the amount of HbF^+ cells and HBG transcripts are low in HUDEP-2 cells. This was the motivation for moving to CD34+ cells. We used flow cytometry as one of three methods to confirm HBG induction, and the confirmation from qPCR, immunoblots, and flow cytometry provides confidence that the induction is real. The flow cytometry data presented representative of two independent repeats. Information of replicates has been added to the figure caption.

Reviewer: For the primary erythroid cell experiments presented in figure 5E-G, were cells from multiple donors tested? What do the error bars in figure 5F represent and how was significance determined? As the western blot quality is not very good, it would be helpful to have some quantification, again from multiple independent donors as biological replicates.

Authors: The data bars represent the mean of 3 replicates and error bars depict the standard deviation. P values were obtained from one-way ANOVA of untreated cells as control. All replicates were from the same donor. To probe the effect on different donors, the experiment was repeated with cells from 2 additional donors. The results, shown in Fig. 5H, reveal the same finding: loss of BCL11A leads to a 3- to 4-fold increase in gamma hemoglobin transcripts. The P values were obtained from two-way ANOVA of untreated cells from a same donor. All the replicates information were added into figure captions as well as SI methods. We elected to use qPCR instead of western blots because western blot is not a particularly quantitative technique. We hope that the reviewer finds that these replicates support the conclusion of the manuscript.

Minor points:

- Page 10, line 12-15 – the authors state that 30% gamma globin transcripts is sufficient to prevent sickling, but that threshold typically refers to HPLC measurements of globin proteins, not transcripts

  We have modified the statement.

- Supplemental Figure S2: I don't think "maturated" is the proper word

  We have removed "maturated".

- "FACS" refers specifically to cell sorting, not analytical flow cytometry

We have restricted the use of “FACS” to instances of cell sorting and have used “analytical flow cytometry” in all other instances.

oc-2022-00998u.R2

Name: Peer Review Information for "A cell-permeant nanobody-based degrader that induces fetal hemoglobin"

Second Round of Reviewer Comments

Reviewer: 2

Comments to the Author

All comments have been addressed adequately

Author's Response to Peer Review Comments:

Thank you. Please find attached a revised version of the main manuscript.
